# Nitrate of Silver in Ophthalmia

**Published:** 1844-01-27

**Authors:** 


NITRATE OF SILVER IN OPHTHALMIA.

M. Velpeau has endeavoured to distinguish the circumstances which should regulate the various modes of employing the nitrate of silver; but as they are all founded on anatomical differences, they seem to us of little use in practice. The employment of the nitrate of silver ought to be founded, not on differences of form, which are very difficult to ascertain, and which are often quite arbitrary, but on differences in the nature of the ophthalmia. There are many kinds of severe ophthalmia in which the nitrate of silver is very efficacious; among these is the purulent variety. In this kind of ophthalmia, which is often connected with some internal disorder, and which is frequently epidemic among children, M. Velpeau proposes to use from one to two parts of the nitrate of silver, dissolved in thirty parts of the vehicle. M. Baron, however, says that in the Foundling Hospital, where this form of ophthalmia is very common among newborn infants, a much stronger solution is found necessary; the proportions being from eight to sixteen parts of the nitrate of silver to thirty of water.—*Lond. Med. Gaz. from Gazette Médicale.*

